# Thyroid Nodules with Nuclear Atypia of Undetermined Significance (AUS-Nuclear) Hold a Two-Times-Higher Risk of Malignancy than AUS-Other Nodules Regardless of EU-TIRADS Class of the Nodule or Borderline Tumor Interpretation

**DOI:** 10.3390/cancers17081365

**Published:** 2025-04-19

**Authors:** Dorota Słowińska-Klencka, Bożena Popowicz, Joanna Duda-Szymańska, Mariusz Klencki

**Affiliations:** 1 Department of Morphometry of Endocrine Glands, Medical University of Lodz, 251 Pomorska Str., 92-213 Lodz, Poland; dorota.slowinska-klencka@umed.lodz.pl (D.S.-K.); bozena.popowicz@umed.lodz.pl (B.P.); 2 Department of Pathomorphology, Medical University of Lodz, 251 Pomorska Str., 92-213 Lodz, Poland; joanna.duda-szymanska@umed.lodz.pl

**Keywords:** thyroid cancer, FNA, EU-TIRADS, Bethesda classification, AUS-nuclear, AUS-other

## Abstract

Background/Objectives: The 2023 revision of the Bethesda System for Reporting Thyroid Cytopathology (BSRTC) simplified the subcategorization of category III into two groups: “AUS-nuclear” and “AUS-other”. The aim of this study was to investigate the risk of malignancy (ROM) of individual BSRTC categories with a particular emphasis on the “AUS-nuclear” and “AUS-other” subcategories and to check whether the low-risk follicular-cell-derived thyroid neoplasm (LRTN) interpretation or EU-TIRADS class of the nodule modify ROM. Methods: The analysis covered the FNA results of 18,225 nodules in 12,470 patients. The rate of malignancy (the upper limit of ROM) was established on the basis of the assessment of 1660 nodules treated surgically in 978 patients. Results: In the broadest variant, with all LRTNs regarded as malignant, the ROM for subsequent categories was as follows: I: 0.4–3.5%, II: 0.1–1.3%, III: 3.8–17.7%, IV: 23.3–27.8%, V: 79.6–90.1%, and VI: 86.3–100.0%. In AUS-nuclear nodules, the ROM was 10.5–28.9%, while in AUS-other nodules, it was 2.2–12.2%. The exclusion of NIFTP or all LRTNs from cancers mainly affected the ROM of AUS-nuclear nodules: 9.4–25.9% or 8.6–23.7%, respectively. EU-TIRADS 5 class increases the ROM in AUS-nuclear nodules to 78.3%, OR: 15.7 and in AUS-other to 40.7%, OR: 6.6. Conclusions: The 2023 BSRTC is a welcome step towards simplification of the way nodules are classified within category III. The AUS-nuclear subcategory is associated with a two-times-higher incidence of malignancy than the AUS-other regardless of LRTN interpretation and EU-TIRADS class of the nodule. The EU-TIRADS 5 class of the nodule is helpful in the identification of category III nodules with a high risk of malignancy.

## Simple Summary

There is a wide range of risk of malignancy (ROM) reported for equivocal categories of the Bethesda classification of thyroid FNA results, especially for category III. It is affected by differences between examined populations, the specificity of the diagnostic center and the adopted method of ROM analysis. Differences in the methodology of the analysis include the way the low-risk follicular-cell-derived thyroid neoplasms (LRTNs) are considered. These neoplasms are morphologically and clinically intermediate between benign and malignant tumors, and their FNA results most often correspond to category III. This category has two subcategories that differ significantly in ROMs-AUS-nuclear and AUS-other. Our research indicates that regardless of how LRTNs are interpreted (as benign or malignant), the ROM of the AUS-nuclear subcategory remains two times higher than that of the AUS-other subcategory. Also, the assessment of the EU-TIRADS class nodule does not change this relationship.

## 1. Introduction

Fine-needle aspiration biopsy (FNA) of the thyroid along with ultrasound imaging (US) is the basic examination used in the diagnosis of thyroid nodules. Since 2008, the results of FNA have been classified according to the Bethesda System for Reporting Thyroid Cytopathology (BSRTC) [1,2,3]. There were two gains related to the implementation of that system. One was the unification of terms used for the cytological diagnoses along with the rules for classification of those diagnoses into particular categories. Another was the determination of the estimated risk of malignancy (ROM) of those categories and its use for recommendation of the optimal management of the thyroid nodule: surgical treatment vs. control FNA (with an optional use of molecular testing) vs. clinical observation. Currently, the Bethesda classification is commonly used despite the fact that there is no agreement between diagnostic centers regarding the ROM of each category. The most prominent differences are reported in the case of equivocal categories: III, IV and V [3,4]. According to the newest edition of the system from 2023, these categories are defined as follows: category III—atypia of undetermined significance (AUS), category IV—follicular neoplasm (FN), and category V—suspicious for malignancy (SM) [3]. Among these, the most controversial is category III, for which there are several fold differences reported both in frequency of occurrence and in ROM [4,5]. This is related to a number of factors, such as the variable epidemiology of thyroid nodules driven by different levels of iodine supply or by the type of diagnostic center (secondary referral level vs. tertiary referral level; oncological center vs. endocrinological center) and various rules for ROM evaluation. One of the important differences in the methodology of ROM assessment is the way the low-risk follicular-cell-derived thyroid neoplasms (LRTNs) are considered. According to the current WHO classification of thyroid tumors, LRTNs include noninvasive follicular thyroid neoplasm with papillary-like nuclear features (NIFTP), hyalinizing trabecular tumors (HTTs) and thyroid tumors of uncertain malignant potential (TT-UMP) consisting of follicular thyroid tumors of uncertain malignant potential (FT-UMP) and well-differentiated tumors of uncertain malignant potential (WD-UMP) [6] These neoplasms are morphologically and clinically intermediate between benign and malignant tumors. As a result, they are treated as benign or malignant for the sake of ROM analyses, or even overlooked in some reports.

Another issue is the quite broad and imprecise definition of category III that leaves a lot of room for individual interpretation. This problem was further aggravated by the changes made to the terms used for the category description [3,7,8]. Category III includes specimens in which the cytomorphological findings are not representative of a benign lesion, yet the degree of cellular, nuclear and/or architectural atypia is not sufficient to render a diagnosis of FN or SM [1,2,3]. Initially, the creators of the Bethesda classification recommended two terms to describe the category intended to be used synonymously, which were to be related to the same ROM: atypia of undetermined significance (AUS) or follicular lesion of undetermined significance (FLUS). The former was intended to be slightly broader and to also refer to cells of non-follicular origin (e.g., lymphoid, parafollicular, parathyroid, etc.) [1]. In practice, in some centers, category III was described by the combined term AUS/FLUS, and some chose one of them (AUS or FLUS), while others used both terms but to denote different forms of atypia. The term AUS was used for the description of nodules with features of nuclear atypia, while the term FLUS was used in the case of nodules with features of architectural atypia. It was quickly observed that the former kind of atypia is related to a twice-higher risk of malignancy than the latter kind [9,10,11]. Gradually, the division of category III into two subgroups began gaining popularity and was officially recognized in the revision of the Bethesda classification in 2018 [12,13,14,15]. In that revision, descriptive language such as “cytologic atypia” (for the features of nuclear atypia) and ‘architectural atypia’ was proposed for the detailed description of category III nodules [2]. Some rarer types were also recognized, such as cases with coexisting cytologic and architectural atypia, lesions composed almost exclusively of Hurthle cells (denoted as Hurthle cell AUS/FLUS), and cases of atypia not otherwise specified. However, contrary to wider practice, the revision upheld the recommendation for the use of terms AUS and FLUS as synonyms that should not be used to denote two distinct interpretations. Only in 2023 did the following revision of the Bethesda classification finally recognize data on the higher ROM of nodules with nuclear atypia in comparison to other types of atypia and the terminology of category III nodules was clarified [3]. That revision discontinued the term “follicular lesion of undetermined significance” to avoid confusion with reporting terminology; henceforth, only the term “AUS” has been recommended. And, consequently, the subcategorization into two groups has been simplified by using two terms: “AUS-nuclear” (previously “cytologic” and “cytologic and architectural”) and “AUS-other”.

Our previous studies showed that the introduction of category III by the Bethesda classification had positive consequences. In particular, it resulted in a decrease in the ROM of category II (benign) and an increase in the negative predictive value (NPV) of this category, as well as an increase in the ROM and the positive predictive value (PPV) of category V [16]. However, those data concerned a period when the division of category III into its subcategories was not routinely used, and the vast majority of diagnoses of that category resulted from the presence of cell architecture atypia typical of follicular lesions. Since 2018, in accordance with the modification of the Bethesda system, we have routinely distinguished cases with various types of atypia in category III, separating the cases with nuclear atypia. The aim of this study was to investigate how the ROM of individual categories of the Bethesda system is shaped in these conditions, with particular emphasis on category III and its current subcategories: AUS-nuclear and AUS-other. We also checked whether the LRTN interpretation or EU-TIRADS class of the nodule significantly modify the ROM.

## 2. Materials and Methods

### 2.1. Patients

The results of thyroid biopsies performed in 2018–2023 in one center were analyzed. The center is of an endocrine profile and is located in a large multi-profile university hospital. The studied population has been covered by an effective, obligatory model of iodine supplementation through iodized salt for almost 30 years [17]. Previously, iodine supplementation was used intermittently, without proper monitoring, and as a result, with low effectiveness. During the examined period, thyroid FNA was performed in 12,470 patients, in whom 18,225 nodules were biopsied. The analysis was limited to the FNA results of those patients who had undergone surgical treatment and for whom the histopathological examination result was known, as that examination was the basis for the assessment of the ROM. Cases in which there was any doubt regarding the topographic equivalence of the biopsied thyroid nodule and the nodule underlying the histopathological diagnosis were excluded from the assessment. Finally, the results of the biopsies of 1660 nodules treated surgically in 978 patients were analyzed, including 138 (14.1%) men and 840 (85.9%) women. The mean age of the patients was 55.4 ± 13.5 (mean ± SD), including women 55.0 ± 13.5 and men 57.4 ± 13.2 (*p* = 0.0546).

### 2.2. Ultrasound, FNA and Histopathological Examinations

The patients were referred for ultrasound imaging (US), FNA and surgical treatment by a physician in the outpatient clinic of endocrinology. All US and FNA examinations of the thyroid were carried out by experienced endocrinologists with a minimum of ten years’ practice. Two ultrasound systems were used for US examinations: the Aloka Prosound Alpha 7 ultrasound system (ALOKA Co., Ltd., Tokyo, Japan) with a 7.5–14 MHz linear transducer (years 2018–2020) and the Aplio a ultrasound system (Canon Medical Systems Europe B.V., Zoetermeer, The Netherlands) with a matrix linear probe 14 L5 (years 2021–2023). The nodules were selected for FNA on the basis of the categorization of the nodule into an appropriate EU-TIRADS category, according to the recommendations of the European Thyroid Association (ETA) [18,19]. The EU-TIRADS defines 5 categories: EU-TIRADS 1 denotes a US examination with no thyroid nodules found; EU-TIRADS 2 (benign category) includes pure cysts and entirely spongiform nodules—FNA is not necessary, unless the patient is scheduled for treatment; EU-TIRADS 3 (low-risk category) includes isoechoic or hyperechoic nodules that show no features of high risk of malignancy—FNA recommended for nodules > 20 mm; EU-TIRADS 4 (intermediate-risk category) includes mildly hypoechoic nodules without any feature of high risk—FNA recommended for nodules > 15 mm; EU-TIRADS 5 (high-risk category) includes nodules that show at least 1 of the following: suspicious shape, irregular margins, microcalcifications, or marked hypoechogenicity—FNA recommended for nodules > 10 mm. FNA was also performed in some nodules smaller than the recommended size for each EU-TIRADS category, following the preferences of the patient or the referring doctor.

During the biopsy, two aspirations of the selected nodule under ultrasound guidance were usually carried out after obtaining informed consent to perform FNA from the patient. Smears were stained with hematoxylin and eosin after fixation in 95% ethanol solution. All biopsy results were formulated in accordance with the version of the Bethesda system in force at the time (i.e., the second or third), which means that in the case of category III, cases with nuclear atypia—currently referred to as AUS-nuclear (formerly cytologic atypia or “architectural and cytologic atypia”)—were distinguished from cases with other types of atypia without nuclear atypia—currently referred to as AUS-other (formerly architectural atypia, oncocytic atypia, not otherwise specified atypia). AUS-nuclear nodules were diagnosed when any abnormal nuclear features such as nuclear enlargement, grooves, prominent nucleoli, abnormal chromatin pattern, alteration in nuclear contour and shape, and/or presence of intranuclear cytoplasmic inclusions were present in an otherwise benign-appearing smear or when a specimen showed nuclear atypia along with limited number of cells. On the other hand, an AUS-other nodule was diagnosed when the specimen showed some degree of architectural atypia (microfollicles, trabeculae or crowding) or predominance of oncocytes or lymphocytic atypia or atypia not otherwise specified but all of that was not sufficient to formulate the diagnosis of neoplasia. Cytological diagnoses were made by a pathologist with over 20 years of experience in the evaluation of cytological and histopathological material from the thyroid gland. Category IV, V and VI BSTRTC diagnoses and some category III diagnoses were formulated on the basis of the agreed opinion of two pathologists (the second was one of two consulting pathologists, also specialized in thyroid cytological diagnostics).

Table 1 shows the distribution of BSRTC categories and EU-TIRADS classes of nodules in the study material. The percentage of nodules of particular EU-TIRADS classes in the examined material was as follows: EU-TIRADS 2–0.8% (14), EU-TIRADS 3–54.6% (907), EU-TIRADS 4–33.7% (560), EU-TIRADS 5–10.8% (179). The percentage of nodules of particular BSRTC categories was as follows: BSRTC I—10.4% (172), BSRTC II—52.6% (873), BSRTC III—24.5% (406), BSRTC IV—2.2% (36), BSRTC V—5.5% (91), BSRTC VI—4.9% (82). The median volume of nodules classified as category II BSRTC was the highest. The median volume of nodules classified as BSRTC category V or VI was similar and lower than that of the other categories (*p* < 0.05). After surgery, a histopathologic evaluation was conducted using standard procedures, and the findings were documented according to the WHO thyroid tumor classification system that was in effect at the time.

### 2.3. Analyses, Statistical Evaluation

The ROM was defined as a range. The lower bound was determined by dividing the number of histopathologically confirmed cancers by the total number of nodules in the analyzed category (regardless of surgical treatment), while the upper bound represented the incidence of cancers among nodules from surgically treated patients only. When more than one FNA of the nodule was performed during the analyzed period, the outcome with the highest category of BSRTC was considered for the calculation of the ROM. In the case of category III, for which control FNA is routinely ordered, the ROM was determined in two variants—when category III was the highest category obtained before surgery and additionally when any FNA outcome was classified as category III (regardless of what was the highest BSRTC category obtained for this nodule during control FNA). A nodule in which various subcategories of category III were recognized on subsequent FNAs was regarded as AUS-nuclear if it was classified into this subcategory at least once. The ROM was calculated in two variants: with or without NIFTP and—more broadly—with all LRTNs regarded as cancers.

Then, we assessed how the upper limit of the ROM of nodules of particular BSRTC category changes when the EU-TIRADS class is taken into account. These analyses were performed in the broadest variant, regarding all LRTNs as malignant lesions. Odds ratios (OR) with relative 95% confidence intervals (95% CIs) were calculated to determine the relevance of the EU-TIRADS class of the nodule on its ROM resulting from the FNA result category.

The statistical analysis was performed with Dell Statistica (data analysis software system), version 13, Dell Inc. (2016), Round Rock, TX, USA. Chi^2^ test was used for the comparison of frequency distributions (with modifications appropriate for the number of analyzed cases). Continuous variables were compared between groups with the use of the Kruskal–Wallis test. The value of 0.05 was assumed as the level of significance.

The study protocol was approved by the local Bioethics Committee. According to the Committee’s approval, neither the patients’ approval nor informed consent for our review of patients’ clinical data and FNA results were needed.

## 3. Results

The ranges of ROM of nodules assigned to particular diagnostic categories of BSRTC are shown in Table 2. In the broadest variant (with all LRTN diagnoses regarded as malignant), the ROM for category I was 0.4–3.5%; category II—0.1–1.3%, category III—3.8–17.7%, category IV—23.3–27.8%, category V—79.6–90.1%, and category VI—86.3–100.0%. In nodules of the AUS-nuclear subcategory, the ROM was 10.5–28.9%, while in AUS-other nodules, it was 2.2–12.2%. When the category III group consisted of nodules with any FNA outcome classified as category III (regardless of what was the highest BSRTC category obtained for this nodule during control FNA), the ROM of nodules of that category was in the range of 4.7–20.6%, with that of the AUS-nuclear subcategory being 13.6–35.1%, and that of the AUS-other subcategory being 2.4–12.8% (Table 3).

The exclusion of NIFTP from the group of cancers, as well as the exclusion of all LRTNs, affected the range of ROM mainly in the case of category III nodules (Table 2). More than 2/3 LRTNs were classified into this category (Table 4). The ROM of category III with the exclusion of NIFTP was 3.5–16.3% for nodules where category III was the highest before the surgical treatment and 4.3–18.9% for nodules where that category was identified in any FNA (Table 2 and 3), with ROM ranges for the AUS-nuclear subgroup of 9.4–25.9% and 12.5–32.5%, and for the AUS-other subgroup of 2.1–11.4% and 2.2–11.7%, respectively. Analogically, the ROM of category III nodules with the exclusion of all LRTNs was 3.2–14.8% for nodules where category III was the highest before the surgical treatment and 4.0–17.6% for nodules where that category was identified in any FNA, including AUS-nuclear, 8.6–23.7% and 11.8–30.5%, and AUS-other, 1.9–10.3% and 2.0–10.6%, respectively.

In all variants of assessment, the malignancy rate of surgically treated nodules of category V or VI (upper limit of ROM) was significantly higher than that of nodules of other BSRTC categories, as well as that of nodules from both subcategories of category III (*p* < 0.0001 in all cases) (Table 2). The malignancy rate of the surgically treated nodules of category II was lower than that of nodules of other categories (*p* < 0.0001).

The malignancy rates of surgically treated AUS-nuclear nodules and category IV nodules were similar and more than 2-fold higher than those of AUS-other nodules. In the assessment variant with LRTNs regarded as malignant lesions, the frequencies were 28.9% and 27.8% vs. 12.2%, respectively, when category III was the highest category of BSRTC before surgery (*p* < 0.0001 and *p* = 0.0113) and 35.1% and 27.8% vs. 12.8%, respectively, when category III was diagnosed in any FNA prior to surgery, regardless of a later higher category (*p* < 0.0001 and *p* = 0.0159) (Table 2 and Table 3). In the case of coexistence of both types of atypia (nuclear atypia and other type atypia) in the AUS-nuclear nodule, the incidence of malignancy was lower than for nodules with nuclear features of atypia only: 13.2% vs. 35.1% when category III was the highest category of preoperative BSRTC (*p* = 0.0116) and 15.0% vs. 42.3% when category III was recognized in any preoperative FNA (*p* = 0.0019) (Table 5). The malignancy rate of oncocytic follicular neoplasms was 37.5% (9 malignancies out of 24 nodules, with LRTNs regarded as malignant) and 33.3% (8 malignancies out of 24 nodules, with LRTNs regarded as benign), while in follicular neoplasms, it was 8.3% (1 out of 12, NS in both variants).

The most common cancer found in the analyzed nodules was PTC (68.1% of all malignant nodules including LRTNs and 73.1% without LRTNs) (Table 4). In more than 3/4 of the cases (76.5%), PTC was classified as category VI and V Bethesda system, and in 19.0% of cases as category III, mainly into its AUS-nuclear subcategory (70.6%). As a result, PTC was found twice as often among cancers in AUS-nuclear nodules as among cancers in AUS-other nodules (61.5% vs. 30.3%, *p* = 0.0082). In addition, postoperative histopathology incidentally revealed 59 PTCs with a size of 0.5 to 7 mm (median 3 mm) in 49 patients. In accordance with the study design, all those incidentally revealed cancers were excluded from the further analysis. The second most common cancer was FTC (8.7% malignant nodules with LRTNs and 9.4% without). FTC was most often classified as category III BSRTC (56.5%) on FNA, mainly into its AUS-other subcategory (76.9%). FTC accounted for 30.3% of cancers in the AUS-other subcategory and 7.7% of cancers in the AUS-nuclear subcategory (*p* = 0.0294). Medullary carcinoma (MTC) accounted for 6.8% and 7.3% of cancers, respectively, and was most often (72.2%) classified as category V BSRTC. Oncocytic thyroid carcionoma (OTC) accounted for 3.8% and 4.1% of cancers, respectively, and was usually (70.0%) classified as category IV BSRTC. The incidences of the other cancers did not exceed 3% and were as follows: anaplastic thyroid carcinoma (ATC)—2.3% (2.4% if LRTNs were not included among malignant nodules); malignant lymphoma (ML)—0.8% (0.8%), secondary tumors 2.7% (2.9%).

Table 6 illustrates the effect of the EU-TIRADS class nodule on the ROM of the nodule as compared to the ROM resulting from the BSRTC category alone. For nodules of category I, II, and III BSRTC, the presence of EU-TIRADS class 5 features resulted in an increase in the rate of malignancy of the nodule as compared to the risk related to the BSRTC category alone. For category I nodules, the ROM increased from 3.5% to 57.1% (*p* < 0.0001), OR (95%CI): 108.7 (14.0–840.6), *p* < 0.0001; for category II—from 1.3% to 31.3%, (*p* < 0.0001), OR (95%CI): 43.1 (12.4–149.7), *p* < 0.0001; for category III—from 17.7% to 58.0% (*p* < 0.0001), OR (95%CI): 9.9 (5.2–18.8), *p* < 0.0001; for subcategory AUS-nuclear—from 28.9% to 78.3% (*p* < 0.0001), OR (95%CI): 15.7 (5.2–47.2), *p* < 0.0001; and for subcategory AUS-other—from 12.2% to 40.7% (*p* < 0.0001), OR (95%CI): 6.6 (2.7–15.8), *p* < 0.0001, respectively. In the case of category IV BSRTC nodules, that increase was close to significant—from 27.8% to 80.0% (*p* = 0.0712), OR (95%CI): 16.7 (1.6–177.5), *p* = 0.0198. A decrease in the incidence of malignancy was observed only in the case of nodules of category III BSRTC that were classified as EU-TIRADS class 3—from 17.7% to 8.6% (*p* = 0.0037), OR (95%CI): 0.7 (0.1–0.5), *p* < 0.0001; specifically for the AUS-nuclear subcategory, it was from 28.9% to 12.9% (*p* = 0.0145), OR (95%CI): 0.2 (0.1–0.5), *p* = 0.0002, and for the AUS-other subcategory, it was from 12.2% to 6.5% (*p* = 0.0834), OR (95%CI): 0.3 (0.1–0.7), *p* = 0.0070.

Among 18 LRTNs, seven each belonged to EU-TIRADS classes 3 and 4, and the remaining four (22.2%) to EU-TIRADS class 5 (3 NIFTP and 1 HTT). The percentage share of EU-TIRADS class 5 in particular types of malignant nodules was as follows: PTC: 58.7% (105), FTC 13.0% (3), OTC: 40.0% (4), MTC: 66.7% (12), ATC: 83.3% (5), ML: 100.0% (2), ST: 71.4% (5).

## 4. Discussion

In the current study, we analyzed how the ROM of all categories of the Bethesda classification is shaped in an endocrinological center that has been dealing with the diagnosis of thyroid nodules for many years. This study was conducted on a population that had been exposed to iodine deficiency in the past, but has been under a mandatory iodine supplementation model for 30 years [17]. Over the years, we observed the effect of the increased iodine supply on the changing epidemiology of thyroid diseases, and consequently on the frequency of individual FNA categories and their ROM. In the 1990s, patients with endemic goiter and with large nonneoplastic nodules in the thyroid gland dominated in our center [16,20]. These factors determined surgical treatment more often than an alarming US image or FNA result. In the initial period of using the Bethesda classification, this trend continued, and the ROM of the indetermined category nodules was significantly lower than in areas rich in iodine [21]. Currently, the situation is quite different. Patients referred for surgical treatment have thyroid nodules, nearly half of which belong to the intermediate- or high-risk EU-TIRADS class, and this rate is similar to that observed in other centers caring for patients with a normal iodine supply [22]. Also, the frequency of malignancy in postoperative histopathology for nodules from particular classes of EU-TIRADS is consistent with the estimated malignancy risk according to ETA guidelines and is as follows: class EU-TIRADS 2–0.0% (estimated malignancy risk 0.0%), EU-TIRADS 3–3.9% (2–4%), EU-TIRADS 4–15.7% (6–17%) and EU-TIRADS 5–78.2% (26–87%) [19]. Under these conditions, the ROM values we currently observe for each FNA outcome category are similar to those presented in the latest modification of the Bethesda classification [3]. In the case of categories III, IV and VI, the incidence of malignancy in postoperative histopathological examination (upper limit of ROM) found in our material was within the recommended ranges, i.e., category III: 17.7% (recommended range: 13–30%), with AUS-nuclear subcategory: 28.9% and AUS-other subcategory: 12.2%; category IV: 27.8% (recommended range: 23–34%); category VI: 100% (recommended range: 97–100%). For categories I and II, the upper limits of the ROM were even slightly lower than expected, 3.5% (5–20%) and 1.3% (2–7%), respectively. In the case of these categories, two factors were probably decisive. The first one is the careful exclusion from the analysis of the incidental thyroid malignancies with a diameter of a few millimeters, which were found outside the nodules subjected to FNA. The second one is the fact that in the case of category I and II BSRTC nodules, it was their size and not their suspicious ultrasound image that was the main reason to perform FNA. In the case of category V, on the other hand, the upper limit of the ROM was higher than expected: 90.1% (67–83%). In this case, the endocrine profile of our center could have been of importance. As we have shown in previous studies, pathologists working in an endocrine center formulate category V diagnosis at a higher level of atypia and, in consequence, less frequently, but they achieve a higher ROM [7]. This may be related to the fact that they assess smears obtained from non-neoplastic lesions more often than their peers from oncological centers. In such smears, features of atypia can be caused by chronic thyroiditis, hyperthyroidism, antithyroid agent use, or radioiodine treatment.

It should be noted that the comparison of data on ROM is hindered not only by factors beyond the control of the diagnostic center, such as iodine supply and patient profile, but also by non-uniform methods of its assessment. ROM analysis can be performed on the sole basis of postoperative histopathological examinations (this is how ROM estimates are presented in subsequent editions of the Bethesda classification) or by additionally using clinical assessment (mainly in the case of category II nodules) or molecular test results (in the case of nodules with indetermined cytology). This extended approach yields lower ROM values compared to the analysis based solely on histopathological assessment. Guzmán-Arocho et al. showed that in the case of category III nodules, the differences in the ROM values can be even higher than 2-fold (ROM of AUS-nuclear nodules: 47% vs. 21%; AUS-other: 23% vs. 6%) [23].

Differences in the methodology of the analysis also concern the way the so-called borderline tumors, which the latest WHO classification of thyroid tumors refers to as LRTNs, are considered [6]. When calculating the ROM, these nodules (especially NIFTP) are regarded in different ways. In some studies, they are classified as benign lesions [24,25], and in other studies as malignant lesions [22,26,27], while other researchers exclude them from their analyses entirely [28,29]. There are also reports where the status of LRTNs is not explained [13,15]. That is why, in our study, we conducted calculations in several variants. In one of them, we analyzed LRTNs together with cancers—considering them as lesions for which surgical treatment is justified. In subsequent variants, we excluded NIFTP cases or all LRTNs from the malignant group. The impact of the way LRTNs are analyzed on the ROM of individual BSRTC categories is not uniform. In our material, more than 2/3 of the nodules that were ultimately determined to be LRTNs were classified as category III BSRTC. Therefore, the biggest differences in the ROM depending on how LRTNs are evaluated were noted in this category, and specifically in its AUS-nuclear subcategory. Similarly, Haaga et al., who investigated the pooled distribution of NIFTP cases in each BSRTC category, revealed that the frequency of NIFTP is highest in category III [30]. Comparable results were also reported by Ruanpeng et al. and Bongiovanni et al. [31,32]. It should be emphasized that the frequency of LRTNs identified in postoperative histopathology varies among geographic areas. This is a consequence of epidemiological differences but also of various diagnostic concepts among institutions and individual pathologists [4]. In particular, the incidence rates of NIFTP in Asia are substantially lower than those observed in North American and European regions (0.5–5% vs. 15–20%) [23,33,34]. In Asia, the most stringent criteria for identifying PTC-type nuclear features are used, which explains why the majority of encapsulated follicular pattern tumors with mild dysplastic nuclear alterations are designated as follicular adenomas [34]. The percentage of LRTNs found in daily practice among nodules considered malignant in our material was 6.8%, and the ratio of NIFTP to PTC was 1:18.

Category III requires a separate comment not only because of the frequent presence of LRTNs, but also for several other reasons. The first reason is related to significant differences in the frequency of category III and in the mutual proportion of its two subcategories that determines the total ROM of category III between diagnostic centers. In our material, the frequency of formulating category III was 10.3%; so, it was in line with the frequency recommended by the creators of the Bethesda classification [3]. The ratio of the AUS-other subcategory (with a lower ROM) to AUS-nuclear (with a higher ROM) was 4:1. The predominance of the frequency of AUS-other subcategories over AUS-nuclear is observed in most publications originating from Europe, while in countries with a high supply of iodine, such as Korea and Japan, the proportion is usually reversed [15,35]. In these countries, cancers are dominated by PTCs (over 90% vs. about 70% in our material) that show features of nuclear atypia [36]. The second important reason for the uniqueness of category III is the fact that it routinely mandates FNA check-ups, which often result in a diagnosis of a different category of BSRTC. Then, a dilemma arises as to which category of BSRTC such nodules should ultimately be classified as. Their inclusion in the highest category ever obtained means that some of the nodules initially assigned to category III are placed in higher categories. This results in the ROM of category III being underestimated. This effect is stronger for the AUS-nuclear subcategory, and the differences in the ROM depending on the way of evaluation reached 6 percentage points in our material. Another bias results from referring only the patients with particularly suspicious nodules of category III for surgery. Such circumstances lead to the overestimation of the ROM of this category (similarly to what we see in the case of category II). The actual ROM of these categories is in a wide range between the lower and upper limits. In our study, 36.3% of AUS-nuclear nodules and 18.0% of AUS-other nodules were treated surgically. Reported data on the frequency of surgery for category III nodules range from more than 10% to over 60%, and significant differences in the frequency of the surgical treatment can be observed between Western practice and Asian practice (40.5% and 29.5%, respectively) [4,25,28]. Asian clinicians may take a more selective surgical approach for patients with category III thyroid nodules.

A more detailed analysis of the AUS-nuclear subcategory showed that this subcategory is also not homogeneous. We found that in nodules with coexisting nuclear atypia and cell architecture atypia, the incidence of their malignancy in the postoperative examination is similar to that observed in the AUS-other subcategory and lower than in isolated nuclear atypia. In the case of the coexistence of architecture atypia with nuclear atypia, the nodules, in some cases, corresponded to follicular adenomas rather than cancers. However, we have not evaluated different types of benign thyroid lesions in detail due to a lack of sufficient data in this regard. Similar observations of a lower ROM of nodules with both types of atypia than nodules with nuclear atypia were reported by Kim et al. 2017 (20% vs. 69%) [14]. On the other hand, Zhao et al., Bagis et al. and Guleria et al. found no significant differences in the ROM of the subgroups in question (89% vs. 84%, 48.2% vs. 38.3% and 59% vs. 50%, respectively) [13,24,37], and Cherella et al. found that ROM was almost two times higher in nodules with both types of atypia than with nuclear atypia in children (75% vs. 41%) [38]. Undoubtedly, this issue requires further research that should include an analysis of different kinds of atypia, with the assessment of intensity of nuclear atypia as well as the identification of detailed architectural atypia patterns [13,24].

In some centers, a similar ROM stratification between particular subcategories is also observed in the case of category IV [35]. This is a consequence of the second edition of the Bethesda classification allowing for the qualification of cases with PTC-mild nuclear features in the background of microfollicular dominant pattern to category IV in order to possibly include cases suspected to be NIFTP [2]. According to the literature, such nodules have a higher ROM than the other cases classified as category IV [27,33,35]. In our center, pathologists hardly ever used that relaxed approach to the morphologic criteria of category IV because that possibility was weakly reflected in the national recommendations for the diagnosis of thyroid nodules only in the year 2022 [39]. These recommendations indicate that category IV should be diagnosed only in cases where the pathologist anticipates the need for surgery. Consequently, category IV was rare in our material, which makes it difficult to conduct a more detailed analysis. FTC was mainly classified as AUS-other nodules, while the majority of cancers in category IV were OTC.

The higher ROM of category III with features of nuclear cell atypia than in other cases in these category raises the question of possible differentiation of further clinical management on this basis [27]. A high ROM speaks in favor of prompt surgical treatment. On the other hand, it is the case of nodules with nuclear atypia features where the effectiveness of control FNA in revealing cancers (including the use of molecular tests) is higher, which makes abandoning this option unjustified. Our previous analyses show that the repeat FNA of AUS-nuclear nodules increases the PPV of this subcategory, while in the case of AUS-other nodules, it increases the NPV and facilitates the resignation from surgical treatment. The addition of ultrasound risk feature assessment further improves the chances for optimal clinical management [40]. We have no experience in the use of commercial molecular tests due to their high costs. But we have shown the usefulness of a non-commercial molecular test based on the analysis of BRAF mutations and expression of selected miRNA combined with repeat FNA and EU-TIRADS assessment [41]. The present study confirms that surgical treatment of category III nodules, especially of AUS-nuclear subtype, is justified when they are classified into the EU-TIRADS 5 class. Among category III nodules, those categorized into the highest EU-TIRADS class had the rate of malignancy increased to almost 80% for AUS-nuclear nodules or 40% for AUS-other nodules. There were only a few studies that considered the distinction between AUS-nuclear and AUS-other nodules when assessing the efficiency of ultrasound risk stratification systems. Most of them showed that such systems are more useful in predicting malignancy for AUS-nuclear rather than the AUS-other subcategory due to a substantial number of cancers other than PTC in the AUS-other subcategory for which TIRADS are less efficient [42,43,44,45,46]. We did not obtain convincing evidence that a low EU-TIRADS class of the nodule should change the clinical management recommended on the basis of the FNA category. There were too few nodules of the EU-TIRADS 2 class. The EU-TIRADS class 3 categorization significantly lowered the ROM of nodules in the AUS-nuclear subcategory to 12.9%. Nevertheless, it remained several times higher than the ROM of the BSRTC category II. For the AUS-other subcategory, the decrease to 6.5% did not reach the level of significance. When it comes to other BSRTC categories, we found a significant increase in the ROM for nodules of class EU-TIRADS 5 from category I BSRTC (up to 57.1%) and II BSRTC (31.3%), which undoubtedly justifies the repeat FNA in such cases. This is concordant with the current guidelines [19].

The limitation of our analysis is the fact that it was performed in one center of a specific character. It is an endocrinological center, specialized in the diagnosis of thyroid nodules, but molecular tests are not routinely used there in patients with an inconclusive FNA result. A part of the population we studied (the elderly) was exposed to iodine deficiency in youth. Our pathologists very rarely used a relaxed approach to the morphologic criteria of category IV as permitted by the second edition of the Bethesda classification, which was a consequence of a slightly different emphasis on this issue in the national recommendations. A limitation of our examination is also the lack of additional verification of histopathological examination results in order to reveal routinely misdiagnosed LRTNs. Nevertheless, the advantage of our study is that it takes into account LRTNs found in daily practice during the ROM analysis and shows how they affect the clinical interpretation of the BSRTC category.

## 5. Conclusions

The Bethesda classification is universal but the wide range of the ROM reported for its particular categories reflects differences between the examined populations (mainly in terms of iodine supply), the specificity of the diagnostic center (endocrinological vs. oncological, secondary referral level vs. tertiary referral level) and the adopted method of ROM analysis (with or without LRTNs, with or without clinical evaluation and molecular test results). Proper clinical interpretation of individual diagnostic categories of BSRTC and a comparison of ROM data between centers require awareness of these factors.

The latest version of the Bethesda classification is undoubtedly a welcome step towards the simplification of the way nodules are classified in category III. The AUS-nuclear subcategory is associated with an incidence of malignancy at least two times higher than that of the AUS-other subcategory regardless of whether or not LRTNs are regarded as malignant for the sake of the analysis or the EU-TIRADS classification is taken into account. The assessment of the EU-TIRADS class of the nodule is more helpful in prompting the referral for surgical treatment than in resignation from rapid thyroid surgery.

## Figures and Tables

**Table 1:** Distribution of BSRTC categories and EU-TIRADS classes of thyroid nodules in the studied material; volume of nodules of particular BSRTC categories.

Bethesda Category	EU-TIRADS [No./%]				All Nodules [No.]	Volume [cm^3^]	
	2	3	4	5		Mean ± SD	Median [Q25–Q75]
I—Nondiagnostic	3/1.7	84/48.8	78/45.3	7/4.1	172	8.7 ± 15.1	2.1 [0.7–10.8]
II—Benign	11/1.3	612/70.1	234/26.8	16/1.8	873	7.1 ± 10.9	2.8 [1.1–8.4] ^a^
III—Atypia of undetermined significance	0	186/45.8	170/41.9	50/12.3	406	7.2 ± 18.9	1.4 [0.5–4.1] ^b^
AUS-nuclear	0	62/45.9	50/37.0	23/17.0	135	7.1 ± 23.7	1.0 [0.4–3.0]
AUS-other	0	124/45.8	120/44.3	27/10.0	271	7.3 ± 16.1	1.8 [0.5–6.3]
IV—Follicular neoplasm	0	10/27.8	21/58.3	5/13.9	36	6.0 ± 10.4	1.3 [0.5–7.3]
V—Suspicious for malignancy	0	9/9.9	37/40.7	45/49.5	91	2.2 ± 5.7	0.5 [0.2–1.2] ^c,d^
VI—Malignant	0	6/7.3	20/24.4	56/68.3	82	3.9 ± 14.4	0.5 [0.2–17] ^c,d^
All	14/0.8	907/54.6	560/33.7	179/10.8	1660	6.9 ± 13.7	2.1 [0.6–6.6]

^a^—*p* < 0.0001 vs. III, V, VI; ^b^—*p* < 0.05 vs. I, V, VI; ^c^—*p* < 0.0001 vs. I; ^d^—*p* < 0.05 vs. IV.

**Table 2:** Ranges of the risk of malignancy in nodules of particular Bethesda system categories; when several FNAs were carried out before the surgery, only the outcome of the highest Bethesda system category was considered.

Bethesda Category	No./% of All Nodules	No./% of Surgically Treated Nodules	Surgery Rate [%]	No. of Malignant Nodules with LRTNs	Lower and Upper Limits of Malignancy Risk with LRTNs [%]	No. of Malignant Nodules Without NIFTP	Lower and Upper Limits of Malignancy Risk Without NIFTP [%]	No. of Malignant Nodules Without LRTNs	Lower and Upper Limits of Malignancy Risk Without LRTNs [%]
I	1509/8.3	172/10.4	11.4	6	0.4–3.5	5	0.3–2.9	5	0.3–2.9
II	14,601/80.1	873/52.6	6.0	11	0.1–1.3	10	0.1–1.1	10	0.1–1.1
III	1874/10.3	406/24.5	21.7	72	3.8–17.7	66	3.5–16.3	60	3.2–14.8
AUS-nuclear	372	135	36.3	39	10.5–28.9	35	9.4–25.9	32	8.6–23.7
AUS-other	1502	271	18.0	33	2.2–12.2	31	2.1–11.4	28	1.9–10.3
IV	43/0.2	36/2.2	83.7	10	23.3–27.8	10	23.3–27.8	9	20.9–25.0
V	103/0.6	91/5.5	88.3	82	79.6–90.1	80	77.7–87.9	79	76.7–86.8
VI	95/0.5	82/4.9	86.3	82	86.3–100.0	82	86.3–100.0	82	86.3–100.0
All	18,225	1660	9.1	263	1.4–15.8	253	1.4–15.2	245	1.3–14.8

LRTNs—low-risk follicular-cell-derived thyroid neoplasms; NIFTP—non-invasive follicular thyroid neoplasm with papillary-like nuclear features.

**Table 3:** Ranges of the risk of malignancy in nodules which were classified into category III of the Bethesda system on any FNA (including cases with a higher category on the repeated FNA).

Bethesda Category	No./% of Nodules	No./% of Surgically Treated Nodules	Surgery Rate [%]	No. of Malignant Nodules with LRTNs	Lower and Upper Limits of Malignancy Risk with LRTNs [%]	No. of Malignant Nodules Without NIFTP	Lower and Upper Limits of Malignancy Risk Without NIFTP [%]	No. of Malignant Nodules Without LRTNs	Lower and Upper Limits of Malignancy Risk Without LRTNs [%]
III	1906	433	22.7	89	4.7–20.6	82	4.3–18.9	76	4.0–17.6
AUS-nuclear	391	151	38.6	53	13.6–35.1	49	12.5–32.5	46	11.8–30.5
AUS-other	1515	282	18.6	36	2.4–12.8	33	2.2–11.7	30	2.0–10.6

LRTNs—low-risk follicular-cell-derived thyroid neoplasms; NIFTP—non-invasive follicular thyroid neoplasm with papillary-like nuclear features.

**Table 4:** Types of thyroid cancers and low-risk follicular-cell-derived thyroid neoplasms found in the nodules of particular Bethesda system categories (considering the highest category of all FNA outcomes of the nodule).

The Highest Bethesda Category	PTC	FTC	OCA	MTC	ATC	ML	ST	LRTNs				All Cases
								NIFTP	HTT	TT- UMP	All LRTNs	
I	4	0	0	0	0	0	1	1	0	0	1	6
II	3	5	0	1	0	1	0	1	0	0	1	11
III	34	13	3	2	3	1	4	6	1	5	12	72
AUS-nuclear	24	3	0	1	2	0	2	4	1	2	7	39
AUS-other	10	10	3	1	1	1	2	2	0	3	5	33
IV	1	1	7	0	0	0	0	0	0	1	1	10
V	62	4	0	13	0	0	0	2	1	0	3	82
VI	75	0	0	2	3	0	2	0	0	0	0	82
All	179	23	10	18	6	2	7	10	2	6	18	263

PTC—papillary thyroid carcinoma. FTC—follicular thyroid carcinoma. OCA—oncocytic carcinoma of the thyroid. MTC—medullary thyroid carcinoma. ATC—anaplastic thyroid carcinoma. ML—malignant lymphoma. ST—secondary tumors. LRTNs—low-risk follicular-cell-derived thyroid neoplasms (NIFTP—non-invasive follicular thyroid neoplasm with papillary-like nuclear features. HTT—hyalinizing trabecular tumors and TT-UMP—thyroid tumors of uncertain malignant potential consisting of follicular thyroid tumors of uncertain malignant potential and well-differentiated tumors of uncertain malignant potential).

**Table 5:** Ranges of the risk of malignancy in nodules in the AUS-nuclear subcategory depending on the coexistence or absence of architectural atypia features in the nodule. The results for nodules for which category III was the highest category before surgery and for nodules that were classified as category III on any FNA (including cases with a higher category on the repeated FNA).

AUS-Nuclear Subcategory	No./% of All Nodules	No./% of Surgically Treated Nodules	Surgery Rate [%]	No. of Malignant Nodules with LRTNs	Lower and Upper Limits of Malignancy Risk with LRTNs [%]	No. of Malignant Nodules Without NIFTP	Lower and Upper Limits of Malignancy Risk Without NIFTP [%]	No. of Malignant Nodules Without LRTNs	Lower and Upper Limits of Malignancy Risk Without LRTNs [%]
Assessing nodules for which category III was the highest category before surgery									
without architectural atypia	287	97	33.8	34	11.8–35.1	30	10.5–30.9	29	10.1–29.9
with architectural atypia	85	38	44.7	5	5.9–13.2	5	5.9–13.2	3	3.5–7.9
Assessing nodules that were classified as category III on any FNA									
without architectural atypia	304	111	36.5	47	15.5–42.3	43	14.1–38.7	42	13.8–37.8
with architectural atypia	87	40	46.0	6	6.9–15.0	6	6.9–15.0	4	4.6–10.0

**Table 6:** Rates of malignancy of nodules in particular categories of FNA outcome as modified by the EU-TIRADS class of the nodule.

Bethesda Category	ROM ^1^ [%]	EU-TIRADS 2		EU-TIRADS 3		EU-TIRADS 4		EU-TIRADS 5	
		Benign/ Malignant Nodules	Rate of Malignancy [%]	Benign/ Malignant Nodules	Rate of Malignancy [%]	Benign/ Malignant Nodules	Rate of Malignancy [%]	Benign/ Malignant Nodules	Rate of Malignancy [%]
I	3.5	3/0	0.0	83/1	1.2	77/1	1.3	3/4	57.1 ^a^
II	1.3	11/0	0.0	611/1	0.2	229/5	2.1	11/5	31.3 ^a^
III	17.7	0	0.0	170/16	8.6 ^b^	143/27	15.9	21/29	58.0 ^a^
AUS–nuclear	28.9	0	0.0	54/8	12.9 ^c^	37/13	26.0	5/18	78.3 ^a^
AUS-other	12.2	0	0.0	116/8	6.5 ^d^	106/14	11.7	16/11	40.7 ^a^
IV	27.8	0	0.0	8/2	20.0	17/4	19.0	1/4	80.0 ^e^
V	90.1	0	0.0	0/9	100.0	6/31	83.8	3/42	93.3
VI	100.0	0	0.0	0/6	100.0	0/20	100.0	0/56	100.0
All	15.8	14/0	0.0	872/35	3.9	472/88	15.7	39/140	78.2

^1^—the upper limit of ROM as estimated without consideration for EU-TIRADS class (FNA rate of malignancy); ^a^—*p* < 0.0001 vs. FNA rate of malignancy; ^b^—*p* = 0.0037 vs. FNA rate of malignancy; ^c^—*p* = 0.0145 vs. FNA rate of malignancy; ^d^—*p* = 0.0834 vs. FNA rate of malignancy; ^e^—*p* = 0.0712 vs. FNA rate of malignancy.

## Data Availability

No new data were created or analyzed in this study. Data sharing is not applicable to this article.

## Abbreviations

The following abbreviations are used in this manuscript:

ACA	anaplastic thyroid carcinoma
AUS	atypia of undetermined significance
AUS-nuclear	atypia of undetermined significance with nuclear atypia
AUS-other	atypia of undetermined significance with other patterns of atypia
BSRTC	Bethesda System for Reporting Thyroid Cytopathology
ETA	European Thyroid Association
EU-TIRADS	European Thyroid Imaging and Reporting Data System
FLUS	follicular lesion of undetermined significance
FN	follicular neoplasm
FNA	fine-needle aspiration biopsy
FTC	follicular thyroid carcinoma
FT-UMP	follicular thyroid tumors of uncertain malignant potential
HTT	hyalinizing trabecular tumors
LRTN	low-risk follicular-cell-derived thyroid neoplasms
MLT	malignant lymphoma
MTC	medullary thyroid carcinoma
NIFTP	noninvasive follicular thyroid neoplasm with papillary-like nuclear features
NPV	negative predictive value
OCA	oncocytic carcinoma of the thyroid
OR	odds ratio
PPV	positive predictive value
PTC	papillary thyroid carcinoma
ROM	risk of malignancy
SM	suspicious for malignancy
ST	secondary tumors
TT-UMP	atypia of undetermined significance with nuclear atypia
US	atypia of undetermined significance with other patterns of atypia
WD-UMP	well-differentiated tumors of uncertain malignant potential
